# Semi-Annual Meeting of the Erie County Medical Society

**Published:** 1869-05

**Authors:** 


### Semi-Annual Meeting of the Erie County Medical Society.

The Semi-Annual Meeting of the Erie County Medical Society will be held on the second Tuesday in June, at the rooms of the Buffalo Medical Association, corner of Main and Eagle streets. It is especially desirable that all physicians holding diplomas from respectable medical colleges, who have not yet become members of this Society, should be present. Membership in the County Society is the only legal criterion of legitimate practice, and without it physicians have no claims to continuance and support from members of the regular profession.

